# Cross-cultural comparison of fertility specific quality of life in German, Hungarian and Jordanian couples attending a fertility center

**DOI:** 10.1186/s12955-016-0429-3

**Published:** 2016-02-24

**Authors:** Réka E. Sexty, Jehan Hamadneh, Sabine Rösner, Thomas Strowitzki, Beate Ditzen, Bettina Toth, Tewes Wischmann

**Affiliations:** Institute of Medical Psychology, Center for Psychosocial Medicine, Ruprecht-Karls University Heidelberg, Bergheimer Strasse 20, 69115 Heidelberg, Germany; Department of Obstetrics and Gynecology, Faculty of Medicine, Jordan University of Science and Technology, King Abdullah University Hospital, Irbid, Jordan; Department of Gynecological Endocrinology and Reproductive Medicine, Women’s Hospital, Ruprecht-Karls University Heidelberg, Heidelberg, Germany

**Keywords:** Infertility, Quality of life, Pronatalism, Cultural differences

## Abstract

**Background:**

Only a few studies have reported cross-cultural comparisons regarding psychosocial consequences of infertility. Differences between societies with different cultural backgrounds were revealed and seemed to be based on the importance of pronatalism. Our aim was to measure cross-cultural differences in fertility specific quality of life of infertile couples in Germany, Hungary and Jordan who attend a fertility center in a cross-sectional study.

**Methods:**

A cross-sectional study was conducted in one fertility clinic in Germany, in five fertility clinics in Hungary and in one fertility clinic in Jordan. Overall 750 couples (252 couples in Jordan, 246 couples in Germany and 252 couples in Hungary) attending the first medical infertility consultation were asked to fill out our questionnaire set. Fertility specific quality of life (FertiQoL) and sociodemographic differences were measured between couples from three countries.

**Results:**

Jordanian couples had the shortest relationship (5.8 ± 4.3 yrs.), though they reported the longest duration of child wish (4.2 ± 3.6 yrs.) and fertility treatments (3.0 ± 3.3 yrs.). The proportion of high education was considerably higher in Jordanian women and men (60 % and 66 %, respectively) compared to the other two samples. First, marked cross-country differences were obtained on Emotional, Mind/Body and Relational subscales of the FertiQoL, indicating that Jordanian couples reported poorer fertility-related quality of life than Germans and Hungarians (*p* < 0.001). After controlling for the sociodemographic and medical variables, a significant difference only in the Emotional domain was observed (*p* < 0.001).

**Conclusions:**

The study revealed only a few cultural based differences in fertility specific quality of life between the couples of the three countries. Thus, infertility counselors should pay attention to psychosocial problems rooted in individual sociocultural aspects of the infertile couple regardless of cultural stereotypes. Further studies should identify sociocultural factors within different subgroups of infertile patients instead of focusing different societies as a whole because intra-cultural psychosocial differences in experiencing infertility seem to be more important for the individual patient than intercultural differences.

## Background

Infertility is a worldwide common health problem affecting approximately 9 % of the population at reproductive age [1]. A large body of research has pointed out that infertility can put significant physical and psychological burden on couples regardless the society they live in [2]. Several studies have indicated that in strongly pronatalist societies, where religious tradition and social norms can put pressure on women and men to become parents [3], suffering from infertility could be a very adverse and stigmatizing experience, especially for women [4, 5], independent of whether they have (a) child/ren already. In their overview, Hynie and Hammer Burns suggested that cultures could be classified from the perspective of *individualism* vs. *collectivism* [6]. Individualist cultures (associated with highly industrialized nations as the United States, the UK or countries in Northern Europe), are defined as cultures in which the goals of individuals take priority over the goals of the in-group. In contrast, collectivist cultures – e.g. China, India, and other East and South Asian countries, followed by African, South American and Middle Eastern countries, − are those in which members of the community place the needs and goals of their important groups ahead of their individual needs ([6], p. 68f). The authors conclude that in collective cultures, social pressure resulting in strained marital and/or social relationships is the predominant stressor in infertile women and men, whereas in individualist countries stressors that involve personal loss might be more significant and profound.

Only a few authors focused on differences in infertility experience in men and women with regard to different cultures. Wischmann and Thorn [7] highlighted, in their review, differences in German and South-African infertile men and found more depressive symptoms among South-African men. Baluch et al. [8] interpreted their results about depression and anxiety in Iranian males reflecting Western results and found differences in impairment of infertile men at follow-up stages. In multicultural countries, some differences were examined in different sub-cultures regarding infertility. Turks living in Germany experienced involuntary childlessness as more stressful than the ethnic Germans [9], and Turkish migrant women in the Netherlands responded to infertility more similarly to women in Turkey than to Dutch women [10].

In order to understand infertility with an integrated approach, cross-cultural comparative studies examining psychosocial consequences of involuntary childlessness are necessary [2]. In the past decades, several infertility-related measurements have been developed and validated in different countries [11–13]. The FertiQoL questionnaire, focusing on fertility specific quality of life (QoL), underwent a long and multinational based developing process and has been used as a disease-specific tool in some European, Asian, and American societies [11, 14–18]. Relevant studies have focused on gender differences in QoL, correlations of fertility specific quality of life with anxiety, depression, and the partners’ infertility-related psychosocial reactions.

In this cross-sectional study, we analyzed whether infertile couples seeking medical help suffer to a greater extent in cultures with a more accentuated pronatalist approach compared to more individualist cultures. Specifically, the study focused on differences in the lives of infertile couples in Jordan and two European countries: Germany and Hungary. Fertility varies among in these countries: in Jordan, the total fertility rate (TFR) in the year 2012 was 3.3 children per women, whereas it was 1.3 in Hungary and 1.4 in Germany [19]. A Jordanian qualitative study on 25 infertile women pointed out that the social and cultural meaning of parenthood had a great impact on how strongly infertile women experience psychosocial disadvantages in their society [5]. We hypothesized that Jordanian infertile couples would report lower levels of quality of life regarding infertility than their counterparts in the European societies, because of supposable stronger pronatalistic cultural values manifested by a high TFR, which might increase the psychosocial burdens of infertility [5, 20].

## Methods

### Sample and recruitment

The study was conducted in Hungary, Germany, and Jordan. In total, one German, one Jordanian and five Hungarian fertility centers participated in the data collection. New patients from the fertility clinics and their partners were enrolled. Both primary and secondary infertile couples were invited by a medical assistant to fill out questionnaires and to sign the consent form while waiting for consultation with a medical doctor. The first two authors were permitted to access medical files of each couple, after the infertility work-up. Data were collected between February 2012 and June 2014. We included couples: if (a) they had been trying to get pregnant with regular, unprotected sexual intercourse at least for a year, but pregnancy had not been established [21]; (b) they showed up for the first time in the participating center; (c) both partners returned the properly filled questionnaires. All Jordanian couples were married; Hungarian and German couples were married or cohabiting, though in both countries a higher proportion was married (75 % and 80 %, respectively).

### Ethical approval

The Institutional Research Board at King Abdullah University Hospital in Jordan, the Scientific and Research Ethics Committee of Health Scientific Board in Hungary, and the Ethics Committee of the Medical Faculty of the Ruprecht-Karls University Heidelberg in Germany provided ethical approval for the study in each country, respectively. The approval included the authors’ access to medical files.

### Measures

The questionnaire consisted of the FertiQoL as well as sociodemographic and medical questions. The medical questions were answered by the first two authors based on the medical files of the couples. Medical data included duration of fertility treatment, type of actual treatment, infertility diagnosis, as well as previous pregnancies and deliveries.

Sociodemographic questions focused on age, education, type of marital status, duration of relationship, and duration of child wish.

The FertiQoL is an internationally developed and validated questionnaire measuring infertility specific quality of life [11]. The Core module of FertiQoL with 24 items was used in this study. The Core module contained four subscales covering emotional (e.g., “Do you fluctuate between hope and despair because of fertility problems?”), mind/body (e.g., “Are your attention and concentration impaired by the thoughts of infertility?”), relational (e.g., “Have fertility problems strengthened your commitment to your partner?”) and social (e.g., “Do you feel social pressure on you to have (or have more) children?”) domains of fertility specific quality of life. A total sum scale was calculated by adding the four subscales and dividing by four. Two general items, (FertiQoL A “How would you rate your health?” and FertiQoL B “Are you satisfied with your quality of life?”, both adapted from the SF-36 Health Survey) of the FertiQoL, were analyzed too. Higher scores indicated better quality of life.

### Data management and analysis

To obtain cross-country differences, we compared sociodemographic and medical variables for the three countries. Continuous variables were analyzed with analysis of variance (ANOVA); categorical variables were compared with chi–square tests. The two parameters, age and education, were analyzed for both men and women separately.

FertiQoL A and B items were transformed into one scale by calculating their means (=FertiQoL AB), and this scale was used as a dependent variable similar to the other four FertiQoL-scales in further analysis. Paired T-tests were performed on all quality of life scales in order to detect gender differences in Germany, Hungary and Jordan. For further analysis, we used the couple as the unit of analysis. For that reason, data was structured so that each row contained the records of both female and male in a couple, in other words, the couples were considered as independent “subjects” [22, 23]. First, repeated-measures analysis of variance (ANOVA) was performed wherein within-subjects factor was the gender, and the country was used as a between-subjects variable. After that, we also performed repeated-measures analysis of covariance (ANCOVA), where sociodemographic and medical variables (age, educational level, duration of relationship, duration of child wish, duration of medical treatment, type of diagnosis and having own child with the partner) were covariates as potential mediators of cross-cultural differences. The significance level for all the analyses was set at *p* ≤0.05. All the analyses were performed using the Statistical Package for the Social Sciences (version 22.0 for Windows, SPSS Inc., Chicago, IL, USA).

## Results

In total, 144 German (response rate: 81 %), 126 Hungarian (response rate: 43 %), and 126 Jordanian (response rate: 81 %) couples agreed to take part in the study. We had to remove data of 21 German couples because questionnaires from one or the other partner were missing. Our final sample contained a total of 750 participants from Jordan (*N* = 252), Hungary (*N* = 252) and Germany (*N* = 246).

Table 1 shows the sociodemographic and medical variables for women and men in the three samples. German women were older than Hungarian and Jordan women, whereas Hungarian men were younger than their counterparts in the other two countries. While the length of relationship was shortest in Jordanian couples (and longest in German couples), Jordanian couples reported the longest duration of wishing for a child and of undergoing through infertility treatment. At the same time, couples already having child(ren), were overrepresented in the Jordanian group (compared to the German and Hungarian groups). In Jordan, both men and women had high levels of education. Hungarian women had high levels of education, too, but not significant compared to the other two samples. With more than 40%, Hungarian couples were more likely to be in the diagnostic process or have a diagnosis of unexplained infertility (compared to about one quarter both in the Jordanian and in the German sample).

**Table 1:** Sociodemographic and medical characteristics in Jordan, Germany and Hungary

	Women				Men			
	Jordan	Germany	Hungary		Jordan	Germany	Hungary	
	(*N* = 126)	(*N* = 123)	(*N* = 126)		(*N* = 126)	(*N* = 123)	(*N* = 126)	
	M (SD)	M (SD)	M (SD)	F	M (SD)	M (SD)	M (SD)	F
Age	30.7 (6.1)	**34.4** (4.6)^**a**^	32.4 (4.9)	15.3*	36.5 (8.19	37.9 (6.2)	**34.5** (5.09^**a**^	8.4*
Duration of relationship	**5.8 (**4.3)^**a**^	**8.4** (5.0)^**a**^	**7.3** (3.7)^**a**^	23.2*				
Duration of child wish	**4.2** (3.6)^**a**^	2.8 (2.1)	2.7 (1.9)	24.1*				
Duration of treatment	**3.0** (3.3)^**a**^	0.6 (0.9)	0.7 (1.5)	99.3*				
	N (%)	N (%)	N (%)	χ^2^	N (%)	N (%)	N (%)	χ^2^
Educational level				52.0*				51.5*
Low	19 (15)	**60** (49)^**a**^	22 (17)		**17** (14)^**a**^	**65** (53)^**a**^	**47** (37)^**a**^	
Medium	24 (19)	**18** (15)^**b**^	40 (32)		33 (36)	**19** (15)^**b**^	39 (31)	
High	**83** (66)^**a**^	45 (36)	64 (51)		**76** (60)^**a**^	39 (32)	40 (32)	
Medical diagnosis				50.9*				
None/unexplained	60 (24)	66 (25)	**110** (44)^**a**^					
Female only factor	76 (30)	76 (31)	80 (32)					
Male only factor	60 (24)	64 (27)	52 (21)					
Mixed factor	56 (22)	42 (17)	**10** (4)^**a**^					
Child with the partner				48.9*				
Yes	**58** (23)^**a**^	14 (6)	14 (6)					

Low educational level: primary and low secondary education, Medium educational level: high secondary education, High educational level: university

*M* mean, *SD* standard deviation, *N* number

* *p* < 0.001

^a^ values marked **bold** significantly differed from values of both other countries at least at level *p* < 0.05

^b^ values marked **bold** significantly differed from values of Hungary at least at level *p* < 0.05

Gender differences (within-subjects differences) on the FertiQoL subscales were observed in the emotional and mind/body quality of life domains; men scored higher on these subscales than women in all of the three samples (Table 2). In Hungary and Jordan, men also reported better social quality of life than women. Thus, gender differences in emotional, mind/body and social domains were confirmed in within-subjects pairwise comparisons of repeated-measures ANCOVAs as well. In our study, the subscales of FertiQoL proved reliable with Cronbach’s alpha ranging between 0.60 and 0.89 for each country.

**Table 2:** Gender (within-couples) differences (calculated with paired t-test) on FertiQoL scales in Jordan, Germany and Hungary

	Jordan			Germany			Hungary		
	Women	Men		Women	Men		Women	Men	
	(*N* = 126)	(*N* = 126)		(*N* = 123)	(*N* = 123)		(*N* = 126)	(*N* = 126)	
FertiQoL Scales	M (SD)	M (SD)	T (df = 125)	M (SD)	M (SD)	T (df = 122)	M (SD)	M (SD)	T (df = 125)
Emotional	52.8 (21.5)	**63.1** (22.7)	4.97*	60.5 (17.6)	**74.4** (16.1)	8.58*	69.0 (16.3)	**81.4** (12.8)	7.53*
α	.77	.81		.79	.84		.77	.67	
Mind/Body	62.7 (23.7)	**72.2** (23.7)	4.55*	72.2 (16.2)	**81.5** (13.8)	7.05*	76.5 (16.7)	**89.7** (10.9)	7.97*
α	.85	.89		.81	.82		.83	.80	
Relational	73.3 (16.8)	75.1 (15.1)	1.45	80.2 (11.6)	77.8 (13.4)	1.94	83.3 (13.4)	85.4 (12.8)	1.72
α	.60	.60		.64	.64		.65	.67	
Social	67.8 (19.7)	**74.8** (18.7)	3.99*	73.0 (17.2)	74.2 (13.9)	0.79	80.3 (13.8)	**86.9** (9.4)	5.32*
α	.73	.75		.75	.67		.64	.61	
FertiQoL AB	3.0 (0.7)	3.0 (0.79)	0.61	2.9 (0.6)	3.0 (0.6)	0.36	2.8 (0.5)	2.9 (0.5)	0.13

FertiQoL AB: calculated as the mean of the scores of the two general questions of FertiQoL (A, B)

Significantly higher scores within-couples (indicating higher QoL for men) are marked **bold**

*M* mean, *SD* standard deviation, *α: Cronbach’s α,* *p < 0.001

Cross-country differences (between-subjects differences) in FertiQoL-subscales are presented in Table 3. First, we found marked differences between the couples of the three countries in emotional, mind/body and relational quality of life domains; Jordanian participants showed the lowest scores, Germans scored higher than Jordanians, and Hungarians scored even higher than the other two groups. After these results were controlled for the relevant sociodemographic and medical variables such as age, educational level, duration of relationship, duration of child wish, duration of medical treatment, type of diagnosis and already having child(ren) as a couple, differences between Germans and Jordanians disappeared in all subscales, except for the emotional QoL. On mind/body and relational FertiQoL-subscales, Hungarian reported better QoL than Germans and Jordanians, even if the results were controlled for sociodemographic and medical variables.

**Table 3:** Cross-country (between-couples) differences in joint FertiQoL-scores of the couples (F-value and PES for repeated-measures ANCOVAs post hoc adjusted with Bonferroni are shown)

FertiQoL Scales	Jordanian couples		German couples		Hungarian couples			
	(*N* = 126)		(*N* = 123)		(*N* = 126)			
	M	SE	M	SE	M	SE	F (df = 2)	PES
Emotional	**59.4** ^**a**^	1.8	**66.4** ^**a**^	1.6	**74.3** ^**a**^	1.4	20.2**	.10
Mind/Body	69.4 ^a^	1.8	75.9^a^	1.4	**81.8** ^**a**^	1.5	12.7**	.06
Relational	75.2 ^a^	1.4	79.1^a^	1.3	**83.3** ^**a**^	1.2	9.0**	.05
Social	73.1	1.6	72.3	1.4	**82.7** ^**a**^	1.3	20.8**	.10
FertiQoL AB	3.1	0.1	2.9	0.1	**2.8** ^**b**^	0.1	4.4*	.02

Values marked **bold**: significant ANCOVA-differences after controlling for the mediating variables (age, educational level, duration of relationship, duration of child wish, duration of treatment, type of diagnosis, child with the partner)

*M* mean, *SE* standard error, *PES* partial eta square

*p < 0.05 **p < 0.01

^a^ significantly differed from values of both other countries at least at level *p* ≤ 0.01

^b^ significantly differed from Jordanian values at least at level *p* ≤ 0.05

We observed differences when country was taken as between-subjects factor: on emotional and social scales, the country accounted for 10 % of the variance of QoL-differences. The impacts of covariates were significant only in some cases. Therefore, age of woman was found to be a significant mediator of differences in domains of mind/body (F(2) = 5.4, *p* < 0.05, PES = 0.01) and social quality of life (F(2) = 3.9, *p* < 0.05, PES = 0.01).

We excluded the possible tendencies for generally scoring extremely high in the Hungarian sample or extremely low in the Jordanian sample because general quality of life (as measured with FertiQoL AB; calculated by the means of FertiQoL A and B items) was rated significantly higher by Jordanian couples than by Hungarian couples.

**Fig. 1:**
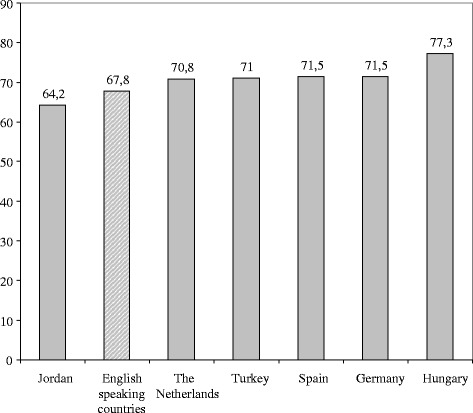
Female scores of FertiQoL Total scale in different published studies. Values: female scores of FertiQoL Total scale. Data were published in recent papers with clinical samples (Boivin et al., 2011; Aarts et al., 2011; Karabulut et al., 2013; Heredia et al., 2013). Combined scores of 291 women and 75 men were reported in the sample of English speaking countries, scores of solely women were reported in Dutch, Turkish and Spanish samples, supplemented by the women’s mean scores of the three samples of our present study

In order to see our results in a broad international context, we collected all recently published data of FertiQoL studies with clinical samples of women from different societies [11, 14, 16–18] and used them to construct a graph of the FertiQoL total scale by country for an extended international comparison (Fig. 1). The results from one study were not included in the graph because of methodological concerns: an analysis of a Taiwanese sample had too poor reliability coefficients, so we did not consider that results [16]. Boivin et al. [11] reported scores of both online and clinical samples, but we included only the scores of the clinical sample (only available for women and men combined). As seen in Fig. 1, fertility specific quality of life especially in German women appears to be in accordance with the data for English speaking, Dutch, Turkish, and Spanish samples. This graph confirmed that FertiQoL can measure similarly the effects in clinic populations of infertile women from different countries.

## Discussion

The main finding of our study is that cross-cultural differences in experiencing primary and secondary infertility related quality of life are not as pronounced as expected. German and Jordanian infertile couples showed quite similar fertility specific quality of life but QoL in Hungarians was high compared to their counterparts from the other two countries. At the same time, the significantly lower quality of life on the emotional FertiQoL-subscale among Jordanian couples (compared with the other two samples) partially supported our hypothesis based on the assumption of a more pronounced pronatalist culture in Jordan. The Jordanian social norm of expecting to have a baby soon after the marriage was obviously reflected in our study, as Jordanian couples exhibited the shortest time living in their marriage. At the same time, they were wishing to have a child and being treated for infertility for the longest time compared to the other two samples.

At the first sight, some cultural differences between the countries were apparent, but the in-group differences were greater (e.g., in the Jordanian sample). In this group, couples exhibited with a broader range of age, relationship duration, duration of child wish, duration of medical treatment. Both partners were located on a broader spectrum in almost all fertility specific quality of life domains (especially on the Emotional and Mind/Body sub-scales), as depicted by the range of standard deviations in the FertiQoL scores of the Jordanian sample (not tested for significance).

Edelmann and Connolly [24] demonstrated that gender stereotypes may be common in studies on the adjustment to infertility and may conceal the real reactions of genders. When the relevant sociodemographic and medical variables were controlled between these countries in the analysis of the couples as a unit (and not of group differences of women and men), the “collectivist” vs. “individualist” dichotomy, while exploring cultural differences, disappeared. This “dichotomy” assumption may lead to incongruent conclusions, and therefore we have to focus on intra-cultural differences at a local level in each country and not on intercultural differences.

Hungarian couples had a higher level of fertility specific quality of life in comparison to Jordanian and German couples. It is an interesting observation in the study which might have several causes: With regards to our results, the GLOBE study [25] (especially with the two sociological scales *gender egalitarianism* and *uncertainty avoidance*) might provide help in interpreting the data. This worldwide study examined societal and organizational attitudes and practices in 62 cultures. *Gender egalitarianism* is defined as “the extent to which a society minimizes gender role differences and gender discrimination” [25]. Hungary stands at the second best position out of the 62 countries studied. This means that practically more life roles are offered to women to fulfill and thus they may not feel under a great pressure to become a mother [26]. According to the definition, *uncertainty avoidance* is “the extent to which members of an organization or society strive to avoid uncertainty by reliance on social norms and bureaucratic practices to alleviate unpredictability” [25]. Hungarians reported on this scale a good tolerance against uncertainty. In this sense, high quality of life regarding fertility problems might reflect that Hungarian infertile men and women could endure better the stress and unpredictability of infertility than Germans or Jordanians. If we consider these explanations, it underlines the necessity of an integrated psychological and sociological approach toward infertility. This points out that viewing fertility specific quality of life as a solely psychological phenomenon is not enough to understand the psychosocial complexity of infertility in different sociocultural settings.

Furthermore, we have to consider other aspects related to the situation of Hungarian couples. The current law in Hungary provides the patients having health insurance with diagnostic procedure—five times through IVF-treatment or six times with insemination treatment—free of charge in fertility clinics under contract with National Health Insurance Fund (OEP). Medications up to 70 % are also supported by OEP [27]. In Germany, health insurance companies cover the costs of diagnosis up to 100 %, although, the costs of treatment and medication are covered, depending on the insurance, at rates of between 50 % and 100 % [28]. In comparison, infertility treatments and investigations are not or are only partly reimbursed by health insurance in Jordan [29]. On the basis of these data, we may assume that financial burdens strengthen psychosocial burdens, at least in Jordan.

At the same time, we have to take into account that the response rate in the Hungarian subgroup was low, so we cannot exclude the possibility that only couples with good adjustment to infertility agreed to participate.

The study has several strengths. To the best of our knowledge, this is the first study to use an internationally validated disease-specific measure assessing fertility-related quality of life in three different countries. The sample sizes are sufficiently large and the study consists of couple-based data.

However, there are some limitations. There might be some sampling bias due to the high rate of refusal to participate in the study in Hungary, and the non-representativeness in Jordan and Germany, where only one university-based clinic was included, respectively. Therefore – and also because we studied convenience samples and not random samples – we cannot clearly postulate that the differences we found between the three samples represent differences between the three countries populations. Furthermore, the Jordanian sample was quite heterogeneous, e.g., duration of treatment ranged from the beginning to long-time therapies or to large time lack between two investigations. In further investigations, sample sizes should be increased by involving more clinics in each country in order to form specific subgroups according to sociodemographic characteristics and medical diagnosis groups. Especially, novel study designs and particular recruitment strategies have to be developed to investigate the experience of infertile individuals not attending fertility clinics. This could also help to distinguish the experience of infertility from the experience of infertility treatment.

For future studies in this field, we also suggest specifying further the sample characteristics focusing on sociocultural aspects in detail and not on psychological variables only. Religiosity, familial and economic status can play important roles in shaping the experience of infertility [30]. Future research could explore if there are larger or smaller differences between countries among men or women, because infertility can have different social meanings for men or women in different countries.

Our results invite infertility counselors to reflect on their own stereotypes regarding the different cultural subgroups of infertile patients. As Hynie and Burns have pointed out, one cannot assume that a cultural norm is descriptive of every member of a given population. “Even countries that appear culturally homogenous are multicultural to some degree, requiring an awareness of diversity issues and the impact of culture on the provision of infertility treatment and infertility counseling” ([6], p. 79). It is recommended that counselors explore the individual couple accurately, attending to their infertility story, their human and financial resources [31], and whether they are supported by the family or the community they belong to, or which specific social expectations they have to meet. Infertility counselors should be attuned to the intra-cultural and inter-individual diversity in the experience of infertility.

## Conclusions

This study highlights that sociocultural differences in experiencing infertility might not be as pronounced as previously assumed in contrast to intra-cultural differences. Our aim was to carry out in a cross-sectional study, at a multinational level, a comparison of psychosocial factors in samples of infertile couples who are seeking medical help using an internationally developed infertility specific measurement. We considered not only cross-country but other possible sociodemographic and medical cultural-related variables (e.g., age, education, duration of child wish, etc.). Cross-country differences were detected in the emotional quality of life domain between Hungary, Germany and Jordan, but not in the other FertiQoL-domains. Thus, we suggest that adjustment to infertility should be examined in the specific cultural context, as well, and the focus of future research on infertility specific quality of life should lie explicitly on subgroup and individual levels within a given society and not on group comparisons between countries alone. Intra-cultural psychosocial differences in experiencing infertility seem to be more important for the individual patient than intercultural differences. These findings underline the hypothesis that infertility is also socially constructed [32] and that its meaning is shaped e.g. by gender ideology, importance of parenthood, treatment options, social policy and cultural stereotypes [33].
